# Combining Autoclaving with Mild Alkaline Solution as a Pretreatment Technique to Enhance Glucose Recovery from the Invasive Weed *Chloris barbata*

**DOI:** 10.3390/biom9040120

**Published:** 2019-03-28

**Authors:** Abraham Kusi Obeng, Duangporn Premjet, Siripong Premjet

**Affiliations:** 1 Department of Biology, Faculty of Science, Naresuan University, Muang, Phitsanulok 65000, Thailand; aobeng@uds.edu.gh; 2 Centre of Excellence in Research for Agricultural Biotechnology, Faculty of Agriculture Natural Resources and Environment, Naresuan University, Muang, Phitsanulok 65000, Thailand; duangpornp@nu.ac.th

**Keywords:** autoclave, *Chloris barbata*, glucose recovery, pretreatment, sodium hydroxide, weed biomass

## Abstract

Developing an optimum pretreatment condition to enhance glucose recovery assessed the potential of *Chloris barbata*, which is a common invasive weed in Thailand, as a feedstock for bioethanol production. *Chloris barbata* was exposed to autoclave-assisted alkaline pretreatment by using different sodium hydroxide (NaOH) concentrations (1% to 4%) and heat intensities (110 °C to 130 °C) that were dissipated from autoclaving. The optimum condition for pretreatment was determined to be 2% NaOH at 110 °C for 60 min. At this condition, maximum hydrolysis efficiency (90.0%) and glucose recovery (30.7%), as compared to those of raw *C. barbata* (15.15% and 6.20%, respectively), were observed. Evaluation of glucose production from 1000 g of *C. barbata* based on material balance analysis revealed an estimated yield of 304 g after pretreatment at the optimum condition when compared to that of raw *C. barbata* (61 g), an increase of five-fold. Structural analysis by the scanning electron microscopy (SEM) and X-ray diffraction (XRD) revealed the disruption of the intact structure of *C. barbata* and an increase in the cellulose crystallinity index (*CrI*), respectively. The results from this study demonstrate the efficiency of using *C. barbata* as a potential feedstock for bioethanol production.

## 1. Introduction

Invasive weeds have been identified as a major environmental and socioeconomic problem due to their ability to infest large areas of arable and non-arable lands, as well as water bodies [1]. However, weed biomass is a promising lignocellulosic feedstock in the economic production of bioethanol [2]. It is a rich source of chemicals and fermentable sugars for bioethanol production and other industrial applications [3]. Weedy plants are very cheap renewable resource and are widely available all over the world. They can grow rapidly on marginal lands under extreme conditions, including drought, low nutrient, and high temperatures, hence requiring no additional economic inputs, such as fertilizers and pesticides [4]. With the current growing interest in cellulosic ethanol production, concerns have been raised regarding the depletion of soil organic carbon pools due to the large-scale removal of crop residues from productive agricultural lands [5]. Excessive removal of crop residue depletes soil carbon pool and negatively impacts the physical, chemical, and biological properties of productive agricultural lands [6]. However, the ability of weedy plants to survive on degraded and abandoned agricultural lands prevents the destruction of productive agricultural lands by reducing the removal of crop residue, making them ideal feedstock for cellulosic bioethanol production. Several weed species, including *Sida acuta*, *Achyranthes aspera* [7], *Eichhornia crasspies*, *Arundo donax*, *Lantana camara*, *Saccharum spontaneum*, and *Mikania mikrantha* [2] have been studied and confirmed as potential feedstock for bioethanol production. Large varieties of weed species grow on both arable and non-arable lands in Thailand. A previous study that was conducted in the northern parts of Thailand explored the potential of several weed species as feedstock in bioethanol production. In particular, *Chloris barbata* was identified as one of the most common weed species growing in the area with the potential as feedstock for bioethanol production [8]. This weedy plant has infested large areas of agricultural and non-agricultural fields in Thailand, significantly contributing to low farm and forest productivity, as well as land degradation [9]. Nevertheless, this weed species is promising feedstock in bioethanol production due to its significant holocellulose content and high dry matter yield [8].

Bioconversion of weed and other lignocellulosic biomass to bioethanol and other high value chemicals involves three main steps, namely; pretreatment, enzymatic hydrolysis, and fermentation [10]. The pretreatment step is very crucial in the efficiency of subsequent downstream processes [11]. It helps to transform the recalcitrant structure of the biomass and expose cellulose fibers. This helps to modify cellulose crystallinity and increase its accessibility to cellulase enzymes [12]. Different pretreatment techniques have specific effects on the physical structure and the chemical composition of lignocellulosic biomass [13]. These physical and chemical variations significantly affect the efficiency of the enzymatic hydrolysis and the fermentation processes [14]. An effective pretreatment technique should be able to enhance the fermentable sugar production and prevent or reduce its degradation. In addition, it should be economical and produce no or less inhibitory by-products [15]. The alkali-based pretreatment technique has attracted much attention of late due to several reasons [16]. It is eco-friendly, non-corrosive, and produces less inhibitory products when compared to acid pretreatment [17]. Alkaline pretreatment breaks the intermolecular ester bonds between the lignin-carbohydrates matrix and modifies the biomass structure, resulting in the solubilization of mainly lignin [18]. This can swell the cellulose, increase its surface area and porosity, as well as decrease its crystallinity to enhance the enzymatic hydrolysis efficiency and increase the yield of fermentable sugars [19]. Sodium hydroxide (NaOH), which is one of the strongest base catalysts, has been identified as a chemical for the effective pretreatment of lignocellulosic biomass [17]. It has been successfully used for the pretreatment of different lignocellulosic biomass, such as hazelnut shells [20], *Phyllostachys edulis* [21], sugarcane bagasse [15], rice husk [19], and rice straw [16]. 

One of the most important chemical pretreatment factors is the supply of heat to facilitate the destruction of bonds between molecules during chemical action [22]. Different heating devices have been used in the pretreatment of lignocellulosic biomass. Moist heat under pressure from the autoclave is one of the most effective heating processes for pretreatment [23]. When compared to other heating sources, the supply of heat with pressure by the autoclave is very effective in rendering the recalcitrant structure of lignocellulosic biomass fragile for chemical action [22]. The moisture present helps to uniformly distribute the heat through the autoclave chamber to improve the efficiency of the heating process [24]. In the present study, an optimum pretreatment condition to enhance glucose recovery from *C. barbata* was established by analyzing the effects of different concentrations of NaOH solutions and autoclave temperatures on hydrolysis efficiency and glucose recovery. Scanning electron microscope (SEM) and X-ray diffraction (XRD) were used to analyze morphological changes in the raw and pretreated *C. barbata*.

## 2. Materials and Methods

### 2.1. Sampling and Processing of Weed Biomass

The *C. barbata* that was used in this study was sampled from Phitsanulok Province, Thailand. The weed biomass was chopped into pieces and then dried in the open air for one week. The dried samples were then milled in a SM 100, Rtsch, Rheinis-che StraBe 36-D-42781 (Haan, North Rhine-Westphalia, Germany) wood miller and passed through 150–300 µm screen. The sieved biomass was stored in tightly closed plastic bags at an ambient temperature until use. 

### 2.2. Analysis of Chemical Composition

The National Renewable Energy Laboratory (NREL, Golden, CO, USA) analytical procedures were used to analyze the chemical composition of the weed biomass before and after pretreatment. The structural carbohydrates, acid insoluble lignin (AIL), and acid soluble lignin (ASL) were determined while following NREL protocol [25]. Acid soluble lignin was analyzed at an absorbance of 205 nm using the ultraviolet-visible (UV-Vis) spectrophotometer (Analytik Jena Specord 40, Analytik Jena AG, Jena, Germany). The high-performance liquid chromatography (HPLC) system (Agilent 1100, Agilent Technologies, Waldbronn, Germany) that was equipped with a G1362A (Agilent Technologies, Waldbronn, Germany) refractive index detector (RID) was used to determine the concentration of monomer sugars. The Bio-Rad Aminex HPX-87P column (Bio-Rad Laboratories, Inc., Hercules, CA, USA) at 80 °C and 20 µL of each sample were used to separate monomer sugars. Filtered HPLC-grade water was used for the elution at a flow rate of 0.6 mL/min. According to NREL method [26], the ash content of the raw weed biomass was analyzed. The ethanol extractives were also evaluated by the NREL protocol [27]. 

### 2.3. Autoclave-Assisted Alkaline Pretreatment

Autoclave-assisted alkaline pretreatment was carried out while using NaOH as a catalyst. *Chloris barbata* (3 g) was added to 24 mL of NaOH in a 125 mL Erlenmeyer flask, mixed well, and tightly sealed with aluminum foil to avoid moisture loss, before heating in the autoclave (TOMY SX-500, Tomy Digital Biology Co., Ltd., Tagara, Nerima-ku, Tokyo, Japan) for 60 min. A two-stage pretreatment process was carried out by varying one factor at a time. In the first stage, the optimum NaOH concentration(s) was determined by pretreating the biomass with 1% (0.25 mol L^−1^), 2% (0.5 mol L^−1^), 3% (0.75 mol L^−1^), and 4% (0.1 mol L^−1^) *w/v* NaOH solutions at a fixed temperature of 120 °C. The optimum pretreatment temperature(s) was then analyzed in the second stage by subjecting the biomass to 2% NaOH solution and then autoclaving at the temperatures of 110 °C, 120 °C, and 130 °C. At the end of the pretreatment process, the slurry was rapidly ice-cooled and then filtered. The solid portion was washed thoroughly with deionized water to attain a neutral pH. The washed autoclave-assisted NaOH pretreated biomass samples were used to further analysis. 

### 2.4. Enzymatic Hydrolysis

Enzymatic hydrolysis of *C. barbata* was carried out as described in a previous study [28]. The raw and pretreated *C. barbata* were hydrolyzed in a 50 mL Erlenmeyer flask with 0.1 g biomass (dry weight) in 10 mL digestion solution. The digestion solution contained 0.05 mol L^−1^ sodium citrate buffer (pH 4.8), 2% (0.3 mol L^−1^) *w/v* sodium azide, and an enzyme mixture of 30 filter paper units (FPU) of cellulase (celluclast 1.5 L, Sigma-Aldrich, St. Louis, MO, USA) plus 60 U β-glucosidase (Oriental Yeast Co., Ltd., Tokyo, Japan) per gram of dry biomass. The reaction was performed at 50 °C for 72 h in a rotary shaker (Innova 4340, New Brunswick Scientific Company, Edison, NJ, USA) at 150 rpm (revolution per minute). The hydrolysates were sampled at 12 h, 24 h, 48 h, and 72 h to analyze the concentration of glucose. The enzymatic hydrolysis efficiency and glucose recovery were calculated by Equations (1) and (2), respectively:

(1) $$\mathrm{HE}\;\left(\%\right)=\frac{\mathrm{Glucose}\;\mathrm{released}\;\left(g\right)}{1.11\times \mathrm{Glucan}\;\mathrm{in}\;\mathrm{initial}\;\mathrm{biomass}\;\left(g\right)}\times 100$$

(2) GR (%)=[SR (%)×glucan (%)×1.11×HE (%)]×100

where HE, GR, and SR represent the hydrolysis efficiency, glucose recovery and solid recovery, respectively. The glucan to glucose conversion factor is 1.11 [28]. 

### 2.5. Surface Morphology of Chloris Barbata

The effect of autoclave-assisted alkaline pretreatment on the surface morphology of *C. barbata* was analyzed using scanning electron microscope (SEM; LEO 1455VP, Zeiss, Gottingen, Germany) at 500× magnification and a beam accelerating voltage of 20 kV. All of the samples were freeze dried and then mounted on aluminum stubs. They were then sputter-coated with gold and observed under the SEM. The representative images were photographed in the study. 

### 2.6. Cellulose Crystallinity Index

X-ray diffraction (XRD) analysis using PANalytical X’pert Pro, PW 3040/60 Diffractometer (Almelo, The Netherlands) characterized the crystalline structure of cellulose in raw and pretreated *C. barbata*. The biomass samples were washed thrice with acetone and then dried at 32 °C. The dried samples were ground to the size of 150 µm mesh screen and scanned at the rate of 0.02° s^−1^ from 2*θ* = 10° to 40°. The CrI was calculated following the relationship below (3):

(3) $$\mathrm{CrI}\;\left(\%\right)=\frac{I_{002}-I_{\mathrm{am}}}{I_{002}}\times 100$$

where *I*_002_ and *I*_am_ represent the maximum intensity peak at 2*θ* = 22.6° and the minimum intensity peak at 2*θ* = 18.6°, respectively. 

### 2.7. Statistical Analysis

All of the analyses were carried out in triplicate. The experimental design was completely randomized in two stages. The first stage (optimal NaOH concentration stage) comprised of five experimental conditions of a single factor (NaOH concentration) with four levels (1%, 2%, 3%, and 4%) plus the control (untreated biomass). The second stage (optimal autoclave temperature stage) was made up of four experimental conditions of one factor (temperature) with three levels (110 °C, 120 °C, and 130 °C), in addition to the untreated biomass control. The data collected were subjected to one-way analysis of variance (ANOVA) using SPSS version 17.0 (SPSS Inc., Chicago, IL, USA). Multiply comparison of treatment means was performed with Tukey’s test at a 5% significance level. The average values were presented with their standard deviations. 

## 3. Results and Discussion

### 3.1. Chloris barbata Characterization

Chemical composition analysis revealed that *C. barbata* (dry weight) is mainly composed of lignin and carbohydrates (Table 1). The carbohydrate portion of raw *C. barbata*, specifically glucan (36.88%), xylan (17.23%), and galactan (1.35%), accounted for approximately 55% of the dry biomass. However, mannose and arabinose were not detected. The total lignin fraction, that is AIL (14.78%) and ASL (4.89%), formed about 20% of the total raw biomass, while other minor components, such as ash and ethanol extractives, accounted for nearly 8% and 7%, respectively. The glucan content of *C. barbata* in this study is in agreement with that of other previously studied invasive weed species, such as; *Chromolaena odorata* (36.2%), *Pennisetum purpureum* (32.0%) [29], *A. aspera* (45.9%), and *S. acuta* (46.9%) [7]. However, the lignin content is an indication for an effective pretreatment process to enhance accessibility to cellulose by cellulase enzymes [30]. Besides enhancing the enzymatic hydrolysis process, the amount of glucose that is recovered is very important and it should be considered in the selection of an effective pretreatment condition(s). Highly severe conditions may decrease the glucose yield and subsequently lead to low bioethanol yield. Consequently, the maximum glucose should be recovered at the optimum pretreatment condition(s) for fermentation into bioethanol [31]. 

### 3.2. Optimization of Sodium Hydroxide Concentration for Pretreatment

**Figure 1:**
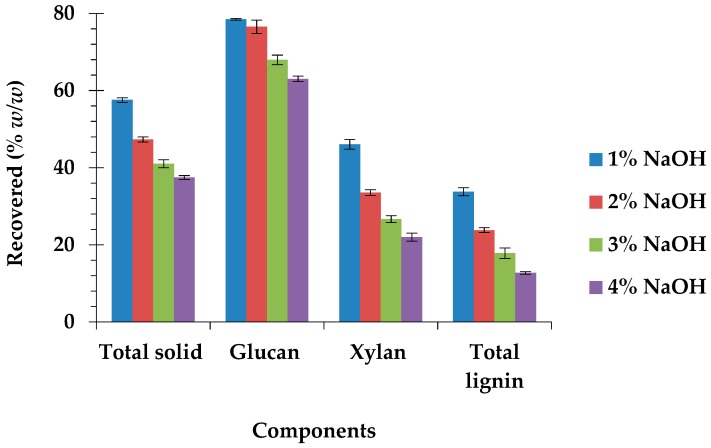
Effect of NaOH concentrations on the chemical composition of *Chloris barbata*.

The efficiency of different NaOH concentrations for the pretreatment of *C. barbata* was evaluated with great emphasis on maximum glucose recovery, in addition to improving enzymatic hydrolysis efficiency. The results (Table 2) showed a highly significant (*p* = 0.0001) decrease in the total lignin content, from 19.67% (raw biomass) to 6.68% (4% NaOH-pretreated biomass), representing approximately 87% lignin removal (Figure 1). Lignin removal significantly (*p* = 0.0001) increased as the NaOH concentration was increased from 1% to 4% (Figure 1), which is consistent with previous studies [15,20,32]. Alkaline pretreatment effectively modifies the structure of lignocellulosic biomass and mainly solubilizes lignin [33]. The NaOH pervade into the interface between lignin and polysaccharides, cleaving the ester bonds between them to promote lignin solubilization as well as the partial degradation of polysaccharides [23]. When compared to the findings in this study, Kang [34] reported the removal of lower lignin content (84.8%) from *Pennisetum* hybrid (*P. americanum* × *P. purpureum*) after pretreatment with the higher NaOH concentration (8%) and autoclave temperature (121 °C). Phitsuwan [35] also extracted lower lignin content (84.1%) from *P. purpureum* with lesser NaOH concentration (2%) and a higher autoclave temperature of 121 °C for 60 min. However, considerably higher lignin content (92.2%) was removed after Lv et al. [17] pretreated sugarcane bagasse with lower NaOH concentration (2%) at a higher autoclave temperature of 121 °C for 60 min. This demonstrates that 4% NaOH at an autoclave temperature of 120 °C is a very effective pretreatment condition in reducing the lignin content in *C. barbata*. 

As in the case of the lignin content, the xylan content was also significantly (*p* = 0.0001) reduced (Table 2) from 17.23% (raw biomass) to 10.12% (4% NaOH-pretreated biomass). Xylan solubilization also significantly increased (*p* = 0.0001) when the NaOH concentration was increased, reaching a maximum of around 80% (Figure 1) when the biomass was pretreated with 4% NaOH concentration. Lignin removal and xylan solubilization caused a highly significant (*p* = 0.0001) increase in the glucan content (Table 2). The glucan content significantly (*p* = 0.0001) increased as the NaOH concentration was increased from 1% to 3%, beyond which there was no significant (*p* = 0.985) increase. The solubilization of lignin and xylan during alkaline pretreatment destroys the complex structure of lignocellulosic biomass and exposes cellulose fibers [21]. Maximum glucan of about 78% was recovered after pretreatment with 1% NaOH concentration, which was not significantly (*p* = 0.236) different from that (about 77%) recovered after 2% NaOH pretreatment. However, there was a highly significant (*p* = 0.0001) increase in glucan solubilization after pretreatment with NaOH concentrations of above 2% (Figure 1). Among the major carbohydrate components in *C. barbata*, glucan was more resistant to degradation by NaOH when compared to xylan. Several studies have reported cellulose to be more resistive to the effect of NaOH than hemicellulose [20,23,32]. The amount of total solid recovered (Figure 1) was significantly reduced with an increase in the NaOH concentration. This may be attributed to the solubilization of lignin, polysaccharides, and other amorphous components during the pretreatment process [36]. Solid loss during pretreatment, especially cellulose, is of great concern, as it significantly affects the amount of sugar that is recovered [37].

Changes in the chemical composition significantly affected cellulose conversion after pretreatment. The efficiency of hydrolysis and glucose recovery of pretreated *C. barbata* was significantly (*p* = 0.0001) improved relative to raw biomass. Maximum hydrolysis efficiency and glucose recovery were obtained after 72 h (Table 2). The highest hydrolysis efficiency (82.26%) was observed in the 4% NaOH-pretreated biomass. However, it was not significantly (*p* = 0.6398) different from that (80.63%) of the 3% NaOH-pretreated biomass, which was also not different (*p* = 0.8595) from that (79.64%) obtained after pretreatment with 2% NaOH concentration. The glucose recovery on the other hand significantly (*p* = 0.0001) increased, reaching a maximum of 24.96% after pretreatment with 2% NaOH concentration. Further increase in the NaOH concentration above 2% lead to a highly significant (*p* = 0.0001) decrease in the glucose recovery. It can be observed from this study that high hydrolysis efficiency did not lead to maximum glucose recovery (Table 2). This phenomenon might be attributed to the significant decrease in total solid, including glucan, at high NaOH concentrations in this study (Figure 1). The optimum pretreatment condition(s) for improving the efficiency of enzymatic hydrolysis may be severe enough to reduce glucose recovery due to a decrease in total solid [31]. Low solid recovery, which significantly affects the sugar recovery during enzymatic hydrolysis, is of great concern, especially under severe pretreatment condition(s) [36]. Xu [37] reported that, although the hydrolysis efficiency increased after NaOH pretreatment of switch grass, there was a significant decrease in total solids, which caused low sugar recovery. The glucose recovery is a very important factor in determining the optimum pretreatment condition(s) of lignocellulosic biomass. However, several researches have focused on the development of optimal pretreatment condition(s) to mainly improve enzymatic hydrolysis efficiency without analyzing the effect that this condition(s) has on the total solid, and hence the glucose recovery. In addition to the high glucose that was recovered after pretreatment with 2% NaOH concentration in this study, the low NaOH concentration is also eco-friendly and economical in the production of second generation bioethanol from *C. barbata*. Therefore, the optimum NaOH concentration of 2% was selected and used for further analysis in the current study. 

### 3.3. Optimization of Pretreatment Temperature

Dissipation of heat with pressure from the autoclave easily destroys the recalcitrant structure of lignocellulosic biomass [22]. However, the intensity of the heating process, among other factors, will depend on the type of lignocellulosic biomass. A perusal of literature showed that autoclave-assisted pretreatment of different lignocellulosic biomass at various temperatures yielded different results [11,23,33,35,38,39]. Therefore, the determination of the optimal temperature for a specific lignocellulosic biomass is very crucial to the success of the pretreatment process.

**Figure 2:**
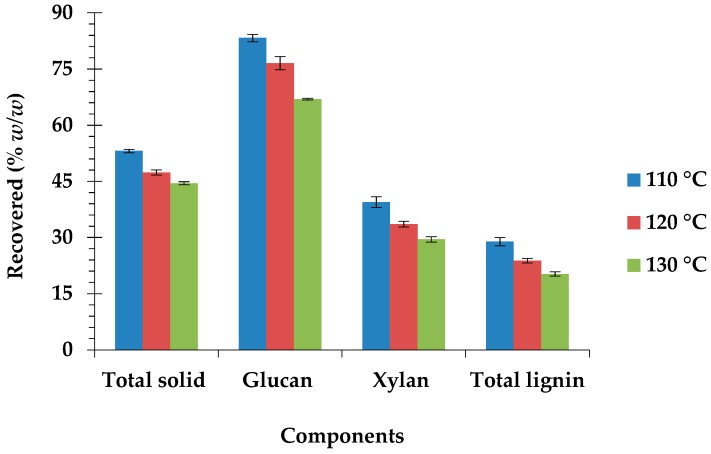
Effect of autoclave temperature on the composition of *C. barbata*.

The optimum autoclave temperature for the pretreatment of *C. barbata* in this study was determined with 2% NaOH concentration at the temperatures of 110 °C, 120 °C, and 130 °C for 60 min (Table 3). According to the results, pretreatment at the various temperatures resulted in highly significant (*p* = 0.0001) variation in the chemical composition of *C. barbata*. Changes in the autoclave temperature also had a highly significant (*p* = 0.0001) effect on the chemical composition. The lignin and xylan contents of *C. barbata* were substantially reduced after pretreatment at different temperatures. Lignin reduction at 110 °C was not significantly (*p* = 0.1635) different from that at 120 °C, but it was significantly (*p* = 0.0040) different from that at 130 °C. Nevertheless, its reduction at the temperatures of 120 °C and 130 °C were the same (*p* = 0.09312, Table 3). Similarly, xylan reduction at 110 °C was not significantly (*p* = 0.21740) different from that at 120 °C, but was significantly (*p* = 0.0040) different from that at 130 °C. However, at the temperatures of 120 °C and 130 °C, xylan reduction was the same (*p* = 0.0704, Table 3). About 71%, 76%, and 80% lignin were removed after pretreatment at 110 °C, 120 °C, and 130 °C, respectively, whiles approximately 61%, 66%, and 71% xylan were solubilized, receptively (Figure 2). The significant solubilization of the amorphous components (mainly lignin and xylan) of *C. barbata* after pretreatment at the various temperatures caused a substantial increase in the glucan content. The glucan content significantly (*p* = 0.0001) increased from 36.88% (raw biomass), reaching a maximum of 59.62% (120 °C). However, further increase in the autoclave temperature up to 130 °C, lead to a highly significant (*p* = 0.0001) decrease in the glucan content (55.53%) when compared to that of the other operating temperatures (110 °C and 120 °C), but not less that of the raw biomass. This may be due to glucan degradation at this high temperature. About 83%, 77%, and 67% glucan contents were recovered at the pretreatment temperatures of 110 °C, 120 °C, and 130 °C, respectively (Figure 2). The total solid recovered at 110 °C, 120 °C, and 130 °C were 53%, 47%, and 44%, respectively (Figure 2). Solid loss is mainly due to lignin removal, as well as xylan and glucan degradation [15]. Pretreatment at the different temperatures significantly improved the hydrolysis efficiency and glucose recovery. The hydrolysis efficiency increased significantly (*p* = 0.0001), from 15.15% (raw biomass) up to 90.03% (110 °C), after which there was a highly significant (*p* = 0.0001) decrease (Table 3). Similarly, significantly (*p* = 0.0001) high glucose recovery (30.70%) was obtained after pretreatment at 110 °C in comparison with that at the other temperatures and raw biomass. 

Therefore, the optimum pretreatment condition for *C. barbata* in this study was determined to be 2% NaOH concentration at 110 °C for 60 min. Maximum glucose recovery of 30.7% was observed at this condition. The highest hydrolysis efficiency of 90.0% was also obtained at this condition, which is higher than that of other lignocellulosic biomass that was pretreated with NaOH, including rice straw (78.7%) [40], *Phoenix canariensis* (87.0%), *Opuntia ficus-indic* (84.5%) [30], and *S. acuta* (82.8%) [41]. It is also obvious from this study that a threshold lignin removal of 71% (Figure 2) in *C. barbata* at the optimum pretreatment condition, among other factors, is enough to allow for cellulolytic enzymes to infiltrate the cell wall. Information about the optimal pretreatment condition(s) is very important in the effective conversion of lignocellulosic biomass to bioethanol [12]. 

### 3.4. Characterization of Raw and Pretreated Chloris barbata by Scanning Electron Microscopy and X-ray Diffraction

**Figure 3:**
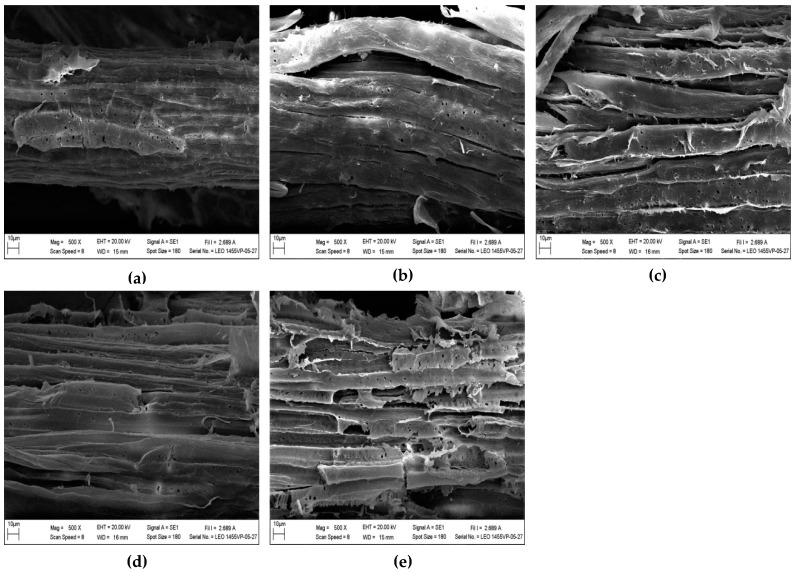
Scanning electron microscope (SEM) images of the (**a**) raw and NaOH-pretreated *C. barbata* with (**b**) 1%, (**c**) 2%, (**d**) 3%, and (**e**) 4% concentrations.

**Figure 4:**
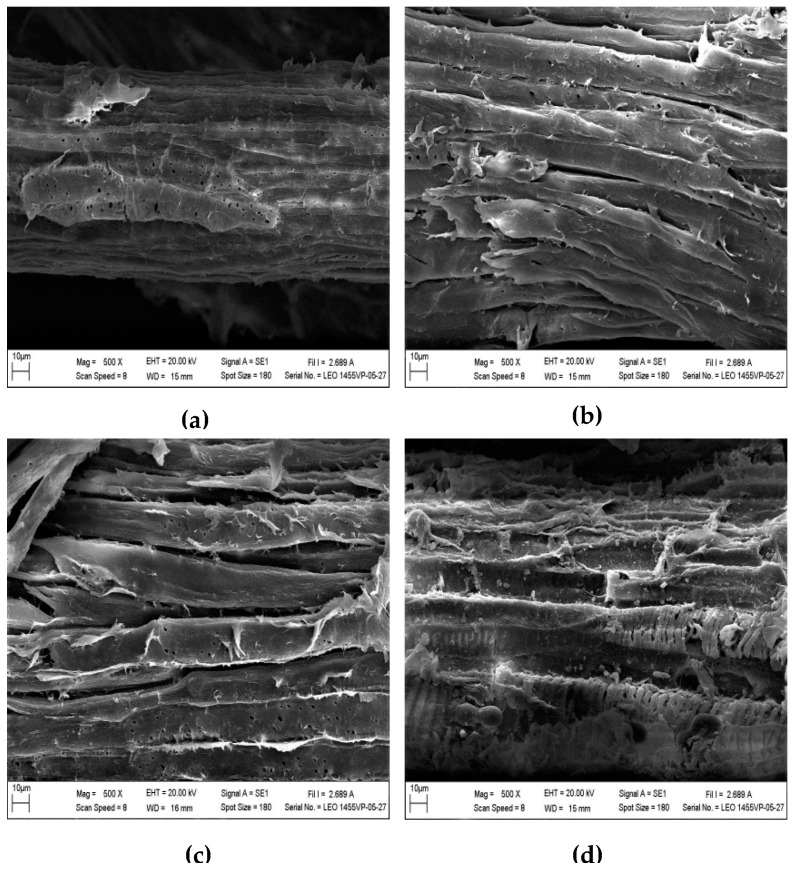
SEM images of the (**a**) raw and autoclave-assisted pretreated *C. barbata* at (**b**) 110 °C, (**c**) 120 °C, and (**d**) 130 °C.

Morphological changes that were induced by autoclave-assisted alkaline pretreatment of *C. barbata* were analyzed to comprehend the structural alterations during the pretreatment process (Figure 3 and Figure 4). The raw *C. barbata* showed a thick smooth surface (Figure 3a and Figure 4a), unlike the pretreated biomass that exhibited uneven surface with cracks and cavities (Figure 3b–e and Figure 4b–d), an indication of the combined effect of the NaOH and steam-pressure from the autoclave. Sodium hydroxide destroys the complex lignocellulosic structure by breaking the bonds linking the lignin-polysaccharide matrix, causing changes in the structure, and thereby increasing the porosity and surface area of the biomass [42]. The combination of steam and pressure from the autoclave generate intense heat to destroy the recalcitrant structure of the lignocellulosic biomass [22]. The surface of the pretreated *C. barbata* was more open exposing the reactive sites of cellulose microfibrils, which enhanced cellulase accessibility. The response of *C. barbata* to different NaOH concentrations and autoclave temperatures was different. In the case of NaOH concentrations, the linkages between the parallel strips of fibers were broken, causing the fiber bundles to loosen and separate (Figure 3b–e). The extensive removal of lignin with an increase in NaOH concentration (Figure 1) caused a higher degree of fiber bundle separation. An increase in the autoclave temperature caused extensive fiber bundles separation. Disintegration of the fibers was observed as the temperature reached a maximum of 130 °C (Figure 4b–d). Fiber separation and disintegration helped to improve access to cellulose to enhance its conversion. 

Cellulose is composed of intra- and inter-molecular hydrogen bonding, which gives it a crystalline structure. Cellulose crystallinity is one of the major causes of biomass recalcitrance [21]. An effective, eco-friendly, and economical pretreatment technology is very important in overcoming biomass recalcitrance [43]. Pretreatment with alkaline is able to modify cellulose structure by destroying the ester bonds, resulting in the solubilization of lignin and hemicellulose. This causes the exposure of cellulose to enzymatic attack [44]. Alkaline pretreatment can also disrupt the crystalline structure of cellulose by destroying the intra- and inter-molecular hydrogen bonding, eventually enhancing its digestibility by cellulase enzymes [12]. Changes in the cellulose structure of *C. barbata* during the pretreatment process were monitored while using XRD. Two main diffraction peaks of around 2*θ* = 15.3° and 22.6°, representing cellulose II and I, respectively, were identified (Figures S1 and S2). An upsurge in the CrI was noticed after pretreatment at the various NaOH concentrations and autoclave temperatures (Table 4). The CrI increased as the concentration of NaOH was increased from 1% to 4%, however, it decreased with an increase in the autoclave temperature, from 110 °C to 130 °C, but not less than that of the raw biomass. The remarkable rise in CrI after pretreatment is in accordance with several earlier researches [2,16,19,21,30]. The rise in CrI is largely due to the decrease in amorphous fractions (mainly lignin and hemicellulose) during pretreatment, exposing the reactive cellulose [35]. An increase in the CrI indicates a rise in cellulose content after pretreatment [2], which is an indication of an effective pretreatment process [42]. It was observed that, at high CrI in the current study, the enzymatic hydrolysis efficiency was greatly improved (Table 2 and Table 3). It is noteworthy to mention that the significant solubilization of lignin and xylan reported earlier (Figure 1 and Figure 2) contributed to enhancing cellulose conversion, although the CrI of the pretreated biomass was not reduced. Similar results have been observed in a number of researches [16,21,22,28,35,42].

### 3.5. Material Balance for the Optimum Pretreatment Condition

**Figure 5:**
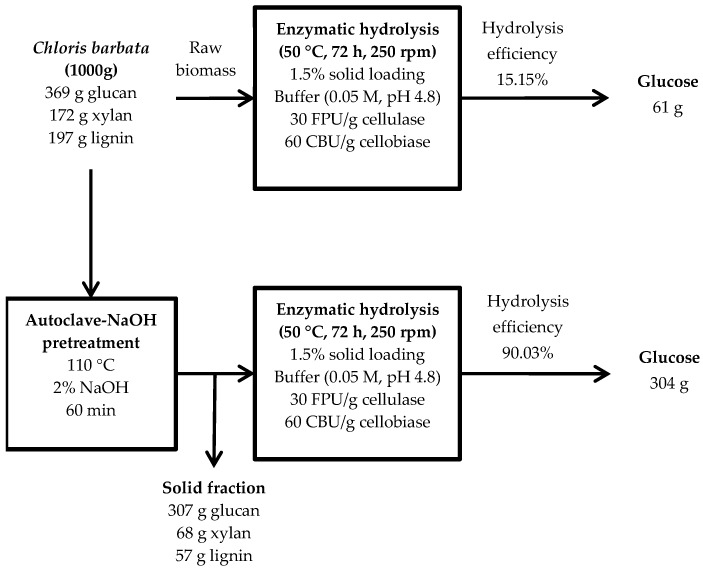
Material balance of glucose recovered from *C. barbata*. FPU: filter paper units; CBU: cellobiase units; rpm: revolution per minute.

The material balance for the optimum pretreatment condition in the current study was performed to evaluate the overall process of converting *C. barbata* into bioethanol. Based on 1000 g of *C. barbata* at the optimum pretreatment condition of 2% NaOH at an autoclave temperature of 110 °C for 60 min, approximately 304 g of glucose can be recovered after enzymatic hydrolysis at the efficiency of 90.03% (Figure 5). However, only about 61 g of glucose can be recovered per 1000 g of raw *C. barbata* after enzymatic hydrolysis at the efficiency of 15.15%. Glucose production from 1000 g of *C. barbata* is estimated to improve by approximately five-fold after pretreatment at the optimum condition in the current study. 

## 4. Conclusions

The synergistic effect of mild alkaline solution and autoclaving was able to effectively destroy the lignin-polysaccharide matrix in *C. barbata*, exposing the cellulose to enzymatic attack to enhance the hydrolysis efficiency and glucose production. Highest hydrolysis efficiency and glucose recovery were obtained at the optimum pretreatment condition of 2% NaOH at 110 °C for 60 min. At this pretreatment condition, glucose recovery was greatly improved by approximately five-fold when compared to the raw *C. barbata*. Based on the findings of this study, *C. barbata* can be confirmed as a potential feedstock for bioethanol production in Thailand.

## Figures and Tables

**Table 1:** Chemical composition of *Chloris barbata*.

Component	% dw (*w/w*)
Glucan	36.88 ± 0.32
Xylan	17.23 ± 0.27
Galactan	1.35 ± 0.08
Ethanol extractive	7.45 ± 0.35
Ash	7.67 ± 0.25
AIL	14.78 ± 0.40
ASL	4.89 ± 0.18
Total lignin	19.67 ± 0.58

% dw represents percentage of dry weight.

**Table 2:** Composition of *C. barbata* pretreated at 120 °C using different concentrations of NaOH.

Composition % dw (*w/w*)	Raw Biomass (% *w/w*)	1 (% *w/w*)	2 (% *w/w*)	3 (% *w/w*)	4 (% *w/w*)
Glucan	36.88 ± 0.32 ^d^	50.30 ± 0.43 ^c^	59.62 ± 0.52 ^b^	61.06 ± 0.41 ^a^	62.03 ± 0.38 ^a^
Xylan	17.23 ± 0.27 ^a^	13.80 ± 0.32 ^b^	12.21 ± 0.24 ^c^	11.19 ± 0.31 ^d^	10.12 ± 0.45 ^e^
ASL	4.89 ± 0.18 ^a^	3.12 ± 0.10 ^b^	2.58 ± 0.13 ^c^	2.17 ± 0.11 ^d^	1.79 ± 0.11 ^e^
AIL	14.78 ± 0.40 ^a^	8.42 ± 0.22 ^b^	7.31 ± 0.37 ^c^	6.36 ± 0.34 ^d^	4.90 ± 0.24 ^e^
Total lignin	19.67 ± 0.58 ^a^	11.54 ± 0.25 ^b^	9.89 ± 0.40 ^c^	8.53 ± 0.44 ^d^	6.68 ± 0.14 ^e^
Hydrolysis efficiency	15.15 ± 0.56 ^d^	61.53 ± 0.86 ^c^	79.64 ± 0.72 ^b^	80.63 ± 0.53 ^ab^	82.26 ± 0.61 ^a^
Glucose recovery	6.20 ± 0.22 ^e^	19.77 ± 0.29 ^d^	24.96 ± 0.33 ^a^	22.43 ± 0.29 ^b^	21.23 ± 0.35 ^c^

The data are average values ± standard deviation (*n* = 3). Average values with identical superscript letters (^a,b,c,d,e^) in the same row are not significantly different at 95% confidence level; dw represent dry weight. Note: 1%, 2%, 3%, and 4% *w/v* NaOH are equivalent to 0.25 mol L^−1^, 0.5 mol L^−1^, 0.75 mol L^−1^, and 0.1 mol L^−1^, respectively. ASL: acid soluble lignin; AIL: acid insoluble lignin.

**Table 3:** Composition of *C. barbata* pretreated with 2% NaOH at different temperatures.

Composition % dw (*w/w*)	Raw Biomass (% *w/w*)	110 °C	120 °C	130 °C
Glucan	36.88 ± 0.32 ^d^	57.84 ± 0.36 ^b^	59.62 ± 0.52 ^a^	55.53 ± 0.44 ^c^
Xylan	17.23 ± 0.27 ^a^	12.78 ± 0.38 ^b^	12.21 ± 0.24 ^bc^	11.42 ± 0.40 ^c^
ASL	4.89 ± 0.18 ^a^	2.76 ± 0.11 ^b^	2.58 ± 0.13 ^bc^	2.28 ± 0.10 ^c^
AIL	14.78 ± 0.40 ^a^	7.92 ± 0.21 ^b^	7.31 ± 0.37 ^bc^	6.67 ± 0.25 ^c^
Total lignin	19.67 ± 0.58 ^a^	10.69 ± 0.32 ^b^	9.89 ± 0.40 ^bc^	8.95 ± 0.31 ^c^
Hydrolysis efficiency	15.15 ± 0.56 ^e^	90.03 ± 0.94 ^a^	79.64 ± 0.72 ^b^	70.29 ± 1.14 ^c^
Glucose recovery	6.20 ± 0.22 ^d^	30.70 ± 0.30 ^a^	24.96 ± 0.33 ^b^	19.27 ± 0.20 ^c^

The data are average values ± standard deviation (*n* = 3). Average values with identical superscript letters (^a,b,c,d,e^) in the same row are not significantly different at 95% confidence level; dw represent dry weight.

**Table 4:** The crystallinity index (CrI) of the raw and autoclave-assisted alkaline pretreated *C. barbata*.

NaOH Concentration (%)	Temperature (°C)	CrI (%)
Raw biomass		32.43
1	120	46.68
2	120	52.27
3	120	56.38
4	120	61.24
2	110	61.53
2	120	52.27
2	130	47.02

## Supplementary Materials

The following are available online at https://www.mdpi.com/2218-273X/9/4/120/s1, Figure S1: XRD patterns of the cellulose-rich fractions of the raw and pretreated *C. barbata* with different NaOH concentrations, Figure S2: XRD patterns of the cellulose-rich fractions of the raw and pretreated *C. barbata* at different autoclave temperatures.
