# Supplementary material for: Combining Autoclaving with Mild Alkaline Solution as a Pretreatment Technique to Enhance Glucose Recovery from the Invasive Weed Chloris barbata
Source: Biomolecules. 2019 Mar 28;9(4):120. doi: 10.3390/biom9040120 (PMC6523731; doi:10.3390/biom9040120)
Supplement: Supplementary file 1 [file biomolecules-09-00120-s001.pdf]

Supplementary Materials

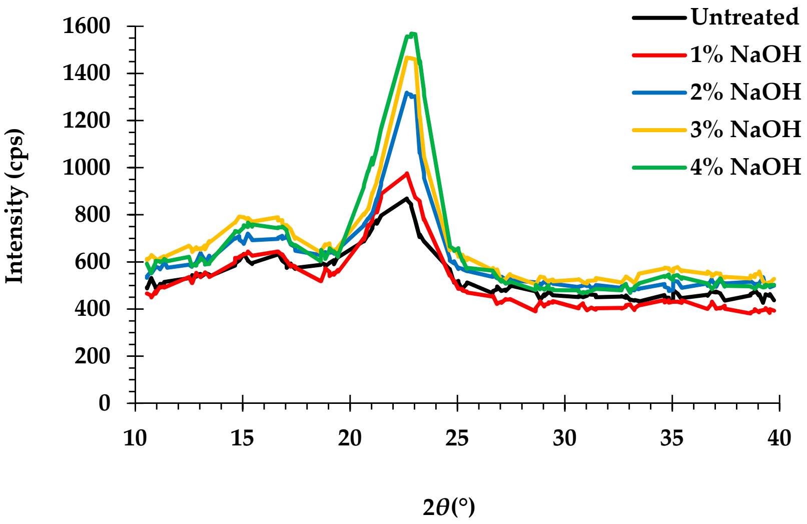

Figure S1. XRD patterns of the cellulose-rich fractions of the raw and pretreated *C. barbata* with different NaOH concentrations.

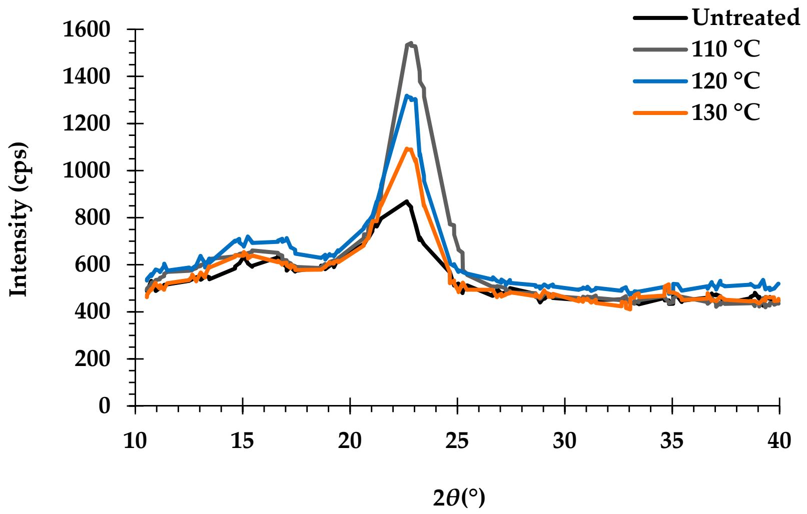

Figure S2. XRD patterns of the cellulose-rich fractions of the raw and pretreated *C. barbata* at different autoclave temperatures.
